# 506. Neonatal CD8^+^ T Cells Demonstrate PD-1 Dependent Impairment Following Human Metapneumovirus Infection

**DOI:** 10.1093/ofid/ofac492.562

**Published:** 2022-12-15

**Authors:** Taylor Eddens, Olivia Parks, Yu Zhang, John Williams

**Affiliations:** UPMC Children's Hospital of Pittsburgh, Pittsburgh, Pennsylvania; UPMC Children's Hospital of Pittsburgh, Pittsburgh, Pennsylvania; UPMC Children's Hospital of Pittsburgh, Pittsburgh, Pennsylvania; UPMC Children's Hospital of Pittsburgh, Pittsburgh, Pennsylvania

## Abstract

**Background:**

Human metapneumovirus (HMPV) is a leading cause of pediatric acute respiratory illness, accounting for 14.2 million acute lower respiratory tract infections and 500,000 hospitalizations per year in children less than 5 years of age globally. Infants and neonates are particularly susceptible to severe HMPV disease. Our prior studies in adult mice demonstrated CD8^+^ T cells were critical for clearance of HMPV infection, although these cells show impaired function due to PD-1:PD-L1 signaling. However, the immune system in the neonatal lung skews towards an anti-inflammatory and tolerogenic response when compared to adults. We therefore sought to explore the roles CD8^+^ T cell and PD-1 signaling in neonatal mice to recapitulate the population at highest risk.

**Methods:**

C57BL/6 or *Pdcd1-/-* pups were infected intranasally with 1.0x10^6^ PFU/g of a clinical isolate of HMPV (TN94-49) on day of life 4-6. Viral burden was assessed via plaque assay and cellular responses were characterized by multispectral flow cytometry.

**Results:**

HMPV infection led to stunted weight gain in pups compared to mock-infected controls. HMPV-infected neonatal mice had a robust innate immune response comprised of neutrophils, CD103^+^ dendritic cells, and interstitial macrophages, all of which demonstrated upregulation of PD-L1. From an adaptive perspective, neonatal mice mounted an antigen-specific CD8^+^ T cell response and resolved HMPV infection by day 8 post-infection. Similar to our prior studies in adult mice, neonatal CD8^+^ T cells upregulated expression of several inhibitory receptors, such as PD-1, LAG-3, and TIM-3. Neonatal CD8^+^T cells demonstrated limited effector cytokine production, such as IFN-γ and granzyme B, following *ex vivo* stimulation with a class I HMPV-specific peptide. *Pdcd1^-/-^* mice (lacking PD-1) also mounted an antigen-specific CD8^+^ T cell response, but had markedly increased production of IFN-γ and granzyme B.

**Conclusion:**

These data demonstrate that PD-1 signaling on CD8^+^ T cells constrains the antiviral response in a novel model of neonatal respiratory viral infection. Understanding the mechanisms that inhibit neonatal anti-viral responses could present opportunities for novel therapeutic strategies for common infections with high morbidity and mortality in neonates and infants.

**Disclosures:**

**John Williams, MD**, GlaxoSmithKline: Advisor/Consultant|Quidel: Advisor/Consultant.

